# Quantitative comparison of manuka and clover honey proteomes with royal jelly

**DOI:** 10.1371/journal.pone.0272898

**Published:** 2023-02-10

**Authors:** Blake W. Paget, Torsten Kleffmann, Kim E. Whiteman, Mark F. Thomas, Chris D. McMahon

**Affiliations:** 1 Hamilton Laboratory, ManukaMed LP, Masterton, New Zealand; 2 Division of Health Sciences, Research Infrastructure Centre, University of Otago, Dunedin, New Zealand; Instituto Butantan, BRAZIL

## Abstract

Royal jelly and honey are two substances produced successively by the worker bee caste. Modern proteomics approaches have been used to explore the protein component of each substance independently, but to date none have quantitatively compared the protein profile of honey and royal jelly directly. Sequential window acquisition of all theoretical fragment-ion spectra mass spectrometry (SWATH-MS) was used to compare protein quantities of bee origin in mānuka and clover honey to royal jelly. Two analysis techniques identified 76 proteins in total. Peptide intensity was directly compared for a subset of 31 proteins that were identified with high confidence, and the relative changes in protein abundance were compared between each honey type and royal jelly. Major Royal Jelly Proteins (MRJPs) had similar profiles in both honeys, except MRJP6, which was significantly more abundant in clover honey. Proteins involved in nectar metabolism were more abundant in honey than in royal jelly as expected. However, the trend revealed a potential catalytic role for MRJP6 in clover honey and a nectar- or honey-specific role for uncharacterised protein LOC408608. The abundance of MRJP6 in mānuka honey was equivalent to royal jelly suggesting a potential effect of nectar type on expression of this protein. Data are available via ProteomeXchange with identifier PXD038889.

## Introduction

The worker bee caste performs hive functions in a temporal manner, which includes secretion of royal jelly by nurse bees, and production of honey via nectar foraging bees. After emergence from the larval stage, nurse bees begin to produce royal jelly as a secretion from the hypopharyngeal and mandibular glands for the development and nutrition of larvae and adult queens. Protein constitutes 10–15% (w/w) of this secretion, of which the MRJPs and, in particular, MRJP1-5 comprise up to 90% of the protein content [1–5]. The central role of royal jelly in larval development [4, 6, 7] and its use as a dietary supplement [8] has driven many proteomics studies. Early work successfully utilized one- and two-dimensional electrophoresis coupled with mass spectrometry to identify a multitude of proteins of bee origin [1, 9–15]. Modern HPLC and high resolution mass spectrometry (HRMS) instruments further increased protein identifications in royal jelly [16] and has allowed detailed insights into post-translation modifications such as glycosylation [17] and phosphorylation [18].

Over a few weeks, nurse bees transition to forager bees. A principal task of the forager bee is to collect nectar, the precursor material of honey. Nectar is stored in a specialized foregut compartment called the crop. In contrast to royal jelly, proteinaceous hypopharyngeal gland secretions of the forager bee become mixed with the plant nectar during ingestion and regurgitation, resulting in the presence of plant proteins in honey, and the overall mass of protein in honey being much lower than in royal jelly. Water evaporation then concentrates the honey to a viscous substance stored in the comb to sustain the energy needs of the hive. Therefore, honey consists mainly of fructose, glucose, and water, with a much lower protein content (mean 0.17% (w/w), range 0.06–0.78%) compared with royal jelly [19, 20].

Honey is categorized based on the nectar type collected. Mānuka honey is produced from nectar collected from flowers of the *Leptospermum scoparium* plant that is indigenous to New Zealand. Mānuka honey has broad spectrum antibacterial activity [21] that is attributed to the presence of methylglyoxal (MGO) [22–24]. Medical grade mānuka honey has greater than 263 mg/kg of MGO (10+ on the UMF grading scale), and honey with concentrations above 572 mg/kg (UMF 16+) are of a premium grade [24, 25]. In contrast, clover honey is produced from nectar collected from *Trifolium* species, which are grown in temperate regions worldwide to fix nitrogen in soil to aid growth of pasture for livestock production [26]. The honey has trace amounts of MGO and has limited antibacterial actions compared to that of mānuka honey [27].

Until recently, only 17 distinct proteins of bee origin had been identified in various honeys using 1-dimensional [28] and 2-dimensional [29–31] gel electrophoresis techniques coupled with mass spectrometry identification. The low yield of information reflected the intrinsically poor resolution of honey proteins after gel separation, and bias toward selecting bands or spots of clear abundance. As with royal jelly, recent studies have utilized high resolution mass spectrometry to identify a large array of proteins of both bee and plant origin in honey with the goal of detecting counterfeit products. In particular, a proteomic analysis of mānuka honey identified 50 proteins of bee origin and 17 mānuka plant proteins [32]. In the same study, further analysis of mānuka nectar confirmed the origin and specificity of candidate peptides from the identified proteins, providing a viable approach to authenticating mānuka honey via peptide profiling. Another comprehensive analysis of 45 different honeys identified 82 proteins of bee origin [33, 34] and provides a means of detecting adulterated honey through peptide identification of foreign amylases.

Here, we aimed to compare the proteomes of the two bee products. Recent research has shown that expression of the major royal jelly proteins is different in the hypopharyngeal glands of nurse and forager bees and most of these differences persist at the protein level [6]. Variations in quantity and composition of other proteins should also exist because collected nectar is stored in the honey crop, which secretes mono- and polysaccharide digestive enzymes into the nectar bolus [35], whereas royal jelly is secreted directly from the glands into the comb. Additionally, the two substances serve completely different functions in the beehive. Taken together these differences should be reflected in different protein profiles for each substance. To investigate and measure potential proteome differences between honey and royal jelly, we employed a label-free quantitation technique termed SWATH-MS. We used two analysis workflows to identify a robust set of bee proteins found in mānuka honey, clover honey and royal jelly. We then compared the quantities of royal jelly proteins to honey proteins to gain insight into protein-level changes between nurse bee and forager bee caste excretions into the respective substances.

## Materials and methods

### Honey and royal jelly samples

Six different clover honeys were purchased from public retail outlets. Pure clover honeys were from Lorimer’s Honey (Hamilton, New Zealand), Pams (Foodstuffs, Auckland, New Zealand) and Airborne Honey (Leeston, New Zealand). Clover blend honeys were from Arataki Honey (Havelock North, New Zealand), Airborne and Mother Earth (Hamilton, New Zealand). Twelve mānuka honeys were sourced from Mother Earth, Red Seal (Auckland, New Zealand), Settlers Honey (Whanganui, New Zealand), and Watson & Son (Masterton, New Zealand). Eight royal jelly samples were analysed. Four, fresh royal jelly samples were a gift from Beaut Bees (Auckland, New Zealand), which were harvested on four different dates in 2018. A single fresh royal jelly sample was purchased from Happy Valley Honey (Auckland, New Zealand). ManukaMed (Masterton, New Zealand) provided royal jelly harvested in the summer of 2016/2017 and frozen at -20°C until analysis. Royal jelly in capsule form was purchased online from Comvita (Paengaroa, New Zealand) and Mānuka Health (Te Awamutu, New Zealand).

### Protein extraction

Honey protein extracts were prepared by diluting 2 g of honey in 2.6 mL phosphate buffered saline (PBS), pH 7.4. Homogenised samples were then dialysed against 300 mL PBS at 4°C with gentle stirring, using 10 kDa molecular weight cut-off SnakeSkin dialysis tubing (Thermo Fisher Scientific). Buffer was changed after 3 h and 6 h, and dialysis continued for a further 16 h. Aliquots of 1 mL were centrifuged 18,000 × g for 5 min and the supernatant transferred to a new tube. Royal jelly protein extracts were prepared by dissolving 80 mg in 4 mL PBS then dialysing as described above. Extracts from capsules were prepared by dissolving 540 mg of capsule material in 4 mL PBS. Insoluble capsule material was removed from the solution by brief centrifugation prior to dialysis. The concentration of protein extracts was determined by BCA assay kit with BSA standards (Merck).

### Mass spectrometry sample preparation

All samples were centrifuged at 16,000 × g for 30 min and 400 μL of supernatant was loaded on a 0.5 mL centrifugal ultrafiltration unit with 3 kDa cut-off membrane (Amicon, Merck) and concentrated down to 20 μL. The retentate was reconstituted in 200 μL of 15% acetonitrile (ACN) and 5 mM tris(2-carboxyethyl)phosphine in 200 mM aqueous triethylammonium bicarbonate (TEAB) for denaturing and reduction of disulphide bonds. After 20 min incubation at room temperature, the volume was reduced by centrifugation to 20 μL and then reconstituted in 200 μL of 15% ACN and 40 mM iodoacetamide in 200 mM aqueous TEAB for alkylation of free thiol groups. After 15 min incubation in the dark at room temperature, buffer was exchanged three times to 200 μL 15% ACN in 200 mM aqueous TEAB. After the last buffer exchange, a small aliquot of each sample was used for protein measurement using the Bradford assay (Bio-Rad). Samples were then concentrated down to 20 μL and supplemented with 5 μg of sequencing grade trypsin (Promega) in 15% ACN in 200 mM aqueous TEAB. Proteins were digested on filters at 37°C overnight and boosted with an additional 2 μg of trypsin in the morning. After an incubation at 37°C for 4 h the samples were dried using a centrifugal vacuum concentrator.

### Shotgun proteomics

To identify the protein component and build a spectral library, an aliquot from each sample of each group (mānuka, clover and royal jelly) was taken and pooled as a representative sample of each group. Each of the three pooled samples was analysed in three technical replicates by data-dependent acquisition using a 5600+ Triple Time-Of-Flight (TOF) mass spectrometer coupled to an Eksigent ekspert nanoLC 415 uHPLC system (AB Sciex). Peptides were loaded onto a 75 μm internal diameter silica emitter tip column packed with Luna (Phenomenex) C18 bead material (3.2 μm, 100 Å) on a length of 20 cm. Peptides were separated by a 90 min LC gradient between mobile phase A (1% ACN, 0.1% formic acid in water) and mobile phase B (0.1% formic acid in 90% aqueous ACN) for mānuka and royal jelly pooled samples and a 60 min LC gradient for the clover and royal jelly pooled samples. The mass spectrometer was operated in data-dependent acquisition (DDA) mode using a mass range of 400–1300 m/z for precursor ion measurement and 100–1600 m/z for fragment ion measurement. The top 15 precursor ion signals per cycle were used for collision-induced dissociation (CID) fragment ion measurements at rolling collision energy. Two repeat measurements of each precursor were allowed during a period of 120 s.

### SWATH-MS

Each individual sample was analysed in technical quadruplicates for mānuka samples and triplicates for clover and royal jelly samples by data-independent acquisition (DIA) using SWATH-MS for protein quantification. The same LC-methods were used as described for the respective DDA analysis to maintain retention time alignment of DIA spectra with the library. For DIA, 33 consecutive fragment ion spectra with variable m/z isolation window sizes were acquired over a mass range of 315–1250 m/z and an ion accumulation time of 100 ms per spectrum resulting in a total cycle time of 3.3 s. The window sizes were calculated based on the precursor ion densities within the different m/z regions of a representative DDA analysis using the SWATH Variable Window Calculator application (AB Sciex). The mass spectrometry proteomics data have been deposited to the ProteomeXchange Consortium via the PRIDE [36] partner repository with the dataset identifier PXD038889.

### Data analysis and library build

For identification of proteins within groups, data from the DIA analyses were processed using DIA-Umpire version 2.0 [37]. Raw files from mānuka and royal jelly 90 min LC runs, and clover 60 min LC runs were converted with ABSCIEX MS Converter (AB Sciex) and msconvert [38]. The files were processed with DIA-Umpire signal extraction module and converted to mzXML format with msconvert. Output files were searched using X!Tandem (2013.06.15.1) with a reference database consisting of all *A*. *mellifera* entries in the National Centre for Biotechnology Information Reference Sequence Database (NCBI RefSeq) collection [39] (comprising 23,491 sequence entries, downloaded from the NCBI site (https://www.ncbi.nlm.nih.gov/) on 20/04/2021) and reverse decoys. Search parameters included trypsin as the cleavage enzyme, with a maximum of two missed cleavages, 50 ppm precursor mass error, and modifications to cysteine and methionine residues. Search outputs were scored and combined with PeptideProphet [40] and ProteinProphet [41]. The ProteinProphet results for each group were processed through the DIA-Umpire quantification module. The protein false discovery rate (FDR) was set at 1% and filtered with a target-decoy method based on protein probability values. Protein abundance was estimated by MS1 peptide intensity-based absolute quantification (iBAQ). Only data were considered where all three technical replicates of at least two biological replicates identified a protein.

For comparative quantification of proteins, raw data of the DDA analyses were processed and searched against the *A*. *mellifera* reference database using ProteinPilot (version 4.5, AB Sciex). Search parameters included trypsin as the cleavage enzyme, biological modifications and single amino acid exchanges were allowed as dynamic modifications, and FDR analysis was enabled. The resulting group file was loaded into the SWATH Acquisition MicroApp 2.0 integrated into PeakView (version 2.2, AB Sciex) to build spectral libraries. For accurate retention time alignment and peak matching, a time window of 12 min and a mass accuracy of 50 ppm were allowed. Peak intensities were then extracted from DIA data using the 6 strongest fragment ions from each of the 10 strongest precursors, at a peptide confidence of ≥99% and FDR threshold for peak matching of ≤1%. Shared peptides were excluded. The extracted area under the curve values were imported into MarkerView (version 1.2, AB Sciex) for data normalisation (based on the total sum of peak intensities).

### Statistical analysis

Data were imported into the statistical computing software R [42] via RStudio (version 1.3.1073, Giant Goldenrod) [43]. Venn diagrams were constructed using the package ‘VennDiagram’ [44]. Normalised label-free quantitation data were tested for variance and normality using Fisher’s test and Shapiro-Wilk test, respectively. A non-parametric bootstrap method [45] was used to calculate 95% confidence intervals for biological replicates and p-values for the difference of means, using 10,000 replicates. Graphs were rendered using the package ‘ggplot2’ [46].

## Results

### Protein identification

**Fig 1:**
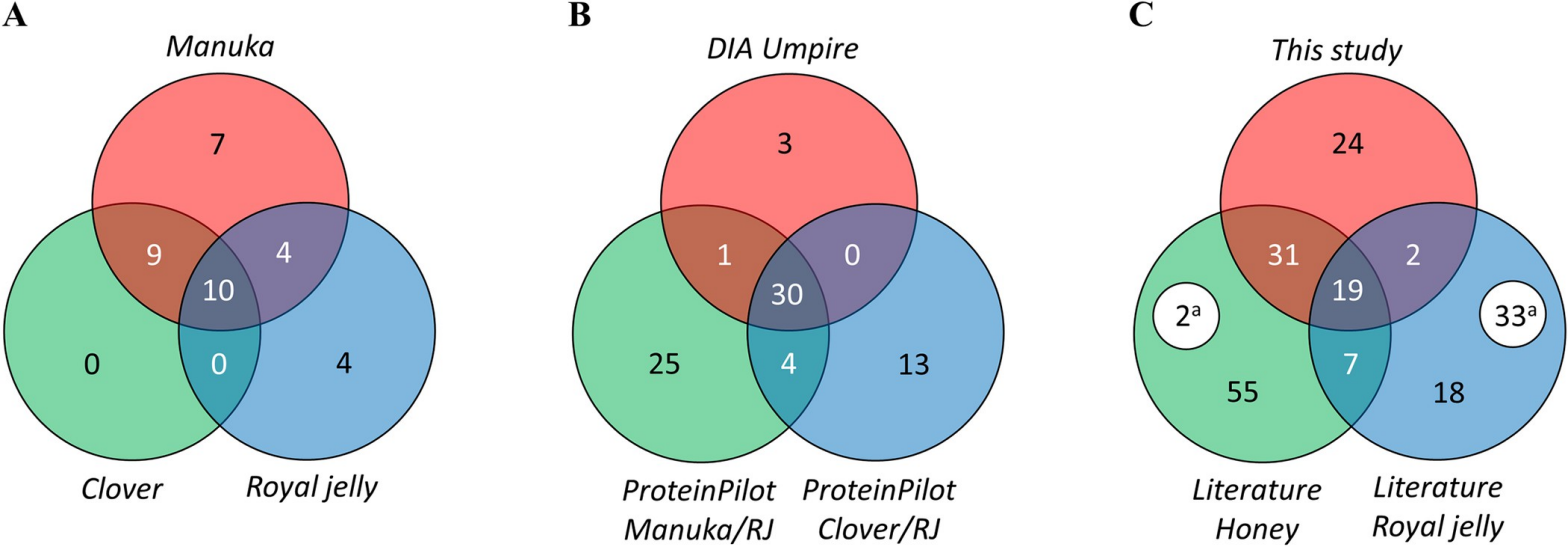
Protein identifications visualised by Venn diagram. (A) Proteins identified in DIA-Umpire by sample group. (B) Proteins identified in DIA-Umpire and the ProteinPilot comparisons of mānuka with royal jelly and clover with royal jelly. (C) Proteins identified in this study and proteins identified in the literature in honey and royal jelly. ^a^ proteins removed from the NCBI RefSeq database.

Signal extraction from DIA data using DIA-Umpire and the X!tandem search engine identified 34 different *A*. *mellifera* proteins across mānuka honey, clover honey and royal jelly sample groups (Fig 1A). A total of 10 proteins were common to all three sample groups. These constituted MRJPs 1–5, MRJP7, an isoform of MRJP5, glucose oxidase, a glucosylceramidase-like isoform, and defensin-1. A total of 16 proteins were found only in honey, 9 of which were common to mānuka and clover honeys, and 7 found only in mānuka honey. None of the identified proteins were unique to clover honey. Four proteins were unique to royal jelly, including apolipophorin-III-like protein, 60S ribosomal protein, lysozyme, and uncharacterised protein (UP) LOC551098. Four proteins were common to royal jelly and mānuka, but not clover honey. These were MRJP9, icarapin-like protein, MRJP2 isoform X1, and uncharacterized protein LOC413627.

In this study there were 76 proteins identified in total (Fig 1B). The ProteinPilot software using the SWATH-MS workflow identified 73 proteins across all samples, encompassing 31 out of 34 proteins identified by the DIA-Umpire/ X!tandem workflow. Two of the three proteins identified only by DIA-Umpire/X!tandem were from royal jelly and included a 60S ribosomal protein and an UP LOC551098, while the remaining glucosylceramidase-like isoform X1 was found in all groups. The SWATH-MS workflows comparing mānuka and clover to royal jelly identified 25 and 13 proteins respectively, that were not found in the other analyses.

The identifications from both workflows were compared to identifications in the literature (Fig 1C). Criteria for inclusion of data from the literature were proteomic analyses of any honey variety or royal jelly, and only proteins identified as being of *Apis mellifera* origin. To create a more concise comparison, NCBI Identical Protein database was used to consolidate entries from the literature that duplicated NCBI RefSeq sequences. UniProtKB/TrEMBL [47] entries with no NCBI accession were aligned using BLAST [48] for nearest hits to NCBI RefSeq sequences. This procedure left a small number of entries sourced from the International Nucleotide Sequence Database Collaboration (INSDC) [49]. While technically different sequences, these entries were very similar isoforms to RefSeq entries. Compared to this consolidated list (see S1 Table) we were able to identify 52 proteins previously identified. There were 19 proteins previously found in both honey and royal jelly. These were MRJPs 1–7, MRJP9, glucose oxidase, alpha-amylase, alpha-glucosidase, hymenoptaecin, defensin-1, icarapin-like protein, apolipophorin-III-like protein, putative glucosylceramidase 4, carboxypeptidase Q, UP LOC408608, and ferritin heavy polypeptide-like 17. A further 31 proteins from this study were previously found in honey alone, and lysozyme and apisimin were previously found in royal jelly alone. There were 24 proteins unique to this study. These included the same two unique royal jelly proteins identified by the DIA-Umpire workflow alone, and 22 identified by the SWATH-MS workflow comprising 10 from clover, 11 from mānuka, and one from both clover and mānuka. In total, 80 proteins were not identified in this study that were in the consolidated list– 55 from honey alone, 18 from royal jelly alone, and 7 from both. Due to database processing, 35 identifications in the literature originating from NCBI RefSeq no longer exist in the database.

The ProteinProphet results and DIA-Umpire quantification module results from the DIA-Umpire workflow are summarised in Table 1. The inclusion of results by DIA-Umpire was strict. Therefore, the error rates reported by ProteinProphet for mānuka, clover and royal jelly identifications at the protein level were 0%, 0.09% and 0%, respectively. Error rates at 1% resulted in 52, 38, and 51 identifications, respectively. The distribution of protein iBAQ values was normal for biological replicates, according to the Shapiro-Wilk test. Across all three groups, MRJP1 was the most abundant protein and had the greatest share of spectrum identifications. In mānuka and royal jelly, the proportion of MRJP1 by abundance was 28.21% and 25.06%, respectively. In clover honey, a much higher than expected proportion for MRJP1 was observed at 42.58%. This was likely an artifact of the much lower coverage of MRJP1 than in the other groups, and the absence of MRJP2 isoform X1. The discrepancy was likely compounded by the shorter chromatography run time used for clover samples and highlights the need for caution when comparing semi-absolute quantification data from different experimental methods. The order of abundance of MRJPs varied in each group but accounted for 91.22%, 95.63% and 95.02% of all peptide intensity in mānuka, clover and royal jelly, respectively.

**Table 1:** DIA-Umpire and ProteinProphet summary statistics.

			Probability			MS1 iBAQ Proportion (% ± SD)			Coverage (%)^a^			Total independent spectra			Share of spectrum identifications (%)		
Accession	Protein	Length (aa)	M	C	R	M	C	R	M	C	R	M	C	R	M	C	R
NP_001011579.1	major royal jelly protein 1 precursor	432	1.00	1.00	1.00	28.21±2.15	42.58±3.09	25.06±4.01	70.8	56.9	73.1	3844	651	928	19.06	26.26	16.51
NP_001011580.1	major royal jelly protein 2 precursor	452	1.00	1.00	1.00	14.17±0.74	15.77±1.08	13.81±2.04	63.7	48.5	79.2	2282	355	634	5.529	12.76	5.94
NP_001011601.1	major royal jelly protein 3 precursor	544	1.00	1.00	1.00	14.72±1.21	11.55±1.5	16.63±2.1	55.5	47.1	59.4	1849	238	607	9.241	9.66	12.05
NP_001011610.1	major royal jelly protein 4 precursor	464	1.00	1.00	1.00	0.78±0.18	1.72±0.21	3.91±1.79	49.4	24.8	44	814	71	250	2.32	2.26	4.31
NP_001011599.1	major royal jelly protein 5 precursor	598	1.00	1.00	1.00	5.79±0.36	6.12±0.7	4.8±1.42	44.8	34.9	47.7	1829	159	367	8.559	5.33	5.27
NP_001011622.1	major royal jelly protein 6 precursor	437	1.00	1.00	-	1.14±0.31	1.26±0.45	-	43.5	29.5	-	476	41	-	2.103	1.8	-
NP_001014429.1	major royal jelly protein 7 precursor	443	1.00	1.00	1.00	3.19±0.5	1.77±0.44	7.87±1.42	40.6	39.1	61.4	907	99	466	4.21	3.55	8.86
NP_001019868.1	major royal jelly protein 9 precursor	423	1.00	-	1.00	0.14±0.03	-	0.45±0.14	14.4	-	5	173	-	40	0.803	-	0.91
XP_026299315.1	major royal jelly protein 2 isoform X1	452	1.00	-	1.00	13.91±0.73	-	12.75±2.66	63.7	-	78.5	2121	-	602	4.983	-	5.17
XP_026299317.1	major royal jelly protein 4 isoform X1	464	1.00	-	-	0.73±0.16	-	-	40.7	-	-	33	-	-	1.58	-	-
XP_026299316.1	major royal jelly protein 5 isoform X1	364	1.00	1.00	1.00	8.43±0.7	14.88±2.09	9.73±5.05	50.3	36.8	51.4	470	63	189	1.982	2.58	1.82
NP_001011608.1	alpha-glucosidase precursor	567	1.00	1.00	-	2.82±0.59	1.53±0.28	-	56.3	42.5	-	1319	132	-	5.911	4.06	-
NP_001011598.1	alpha-amylase precursor	493	1.00	1.00	-	0.33±0.08	0.26±0.08	-	41	17.8	-	585	65	-	2.234	1.92	-
XP_393208.1	putative glucosylceramidase 4	511	1.00	1.00	-	0.16±0.04	0.34±0.09	-	16	5.3	-	184	20	-	0.9	0.72	-
XP_006570610.1	glucosylceramidase-like isoform X2	511	1.00	1.00	1.00	0.07±0.03	0.23±0.08	1.59±0.67	13.3	3.7	20.5	35	15	97	0.125	0.48	1.28
XP_393161.3	lysozyme	153	-	-	1.00	-	-	0.44±0.16	-	-	17	-	-	24	-	-	0.31
NP_001011574.1	glucose oxidase	615	1.00	1.00	1.00	1.06±0.17	0.5±0.31	0.08±0.04	30.4	19.8	14.3	733	54	51	3.381	1.58	0.63
XP_625189.3	laccase-5	645	1.00	0.99	-	0.07±0.02	0.11±0.05	-	9.5	2	-	140	14	-	0.482	0.6	-
XP_003251148.1	glucose dehydrogenase	625	1.00	1.00	-	0.13±0.03	0.11±0.05	-	23.2	7.4	-	263	15	-	1.295	0.43	-
XP_006567694.1	glucose dehydrogenase isoform X1	636	1.00	-	-	0.01±0	-	-	12.1	-	-	30	-	-	0.093	-	-
XP_026299638.1	lipase member H-A	304	1.00	0.99	-	1.13±0.26	0.73±0.25	-	9.5	7.2	-	131	16	-	0.59	0.74	-
XP_016770320.2	esterase B1	553	1.00	1.00	-	0.28±0.09	0.36±0.12	-	32.4	8.7	-	222	28	-	1.142	0.91	-
XP_006560620.1	venom serine protease Bi-VSP	353	1.00	-	-	0.11±0.02	-	-	25.5	-	-	227	-	-	0.973	-	-
XP_006558902.1	carboxypeptidase Q	479	1.00	-	-	0.08±0.02	-	-	20.5	-	-	117	-	-	0.534	-	-
XP_006563421.1	chymotrypsin inhibitor-like	74	1.00	-	-	0.41±0.18	-	-	20.3	-	-	70	-	-	0.35	-	-
NP_001011616.2	defensin-1 preproprotein	95	1.00	0.99	1.00	0.09±0.03	0.78±0.16	0.29±0.29	47.4	9.5	33.7	13	9	22	0.049	0.39	0.4
NP_001107670.1	apolipophorin-III-like protein precursor	193	-	-	1.00	-	-	2.47±0.83	-	-	59.1	-	-	74	-	-	1.08
XP_006558672.1	60S ribosomal protein L6	274	-	-	0.96	-	-	0.53±0.41	-	-	2.6	-	-	9	-	-	0.2
XP_006560325.2	LOC102655185 isoform X1 ^b^	1203	1.00	-	-	0.1±0.04	-	-	3.6	-	-	112	-	-	0.267	-	-
XP_016769016.1	chitinase-like protein Idgf4 isoform X2	438	1.00	-	-	0.02±0.01	-	-	10	-	-	60	-	-	0.14	-	-
NP_001012431.1	icarapin-like precursor	223	1.00	-	1.00	0.22±0.06	-	1.46±0.54	25.1	-	21.9	123	-	66	0.451	-	1.36
XP_026298038.1	LOC413627 ^b^	654	1.00	-	1.00	0.11±0.05	-	0.15±0.09	15.7	-	13.5	173	-	49	0.552	-	0.48
XP_397512.1	LOC408608 ^b^	181	1.00	1.00	-	1.68±0.34	0.18±0.12	-	51.4	47.5	-	520	65	-	2.448	2.39	-
XP_623499.1	LOC551098 ^b^	286	-	-	0.99	-	-	0.88±0.25	-	-	2.4	-	-	7	-	-	0.16

M, mānuka sample group; C, clover sample group; R, royal jelly sample group.

^a^ Signal sequences of secreted proteins were not excluded from analyses.

^b^ Uncharacterised proteins.

Metabolic enzymes made up the remainder of protein abundance for both honeys, many of which were absent from royal jelly. Conversely, the remaining royal jelly proteins were poorly represented or not found in the honey samples. These included glucoslyceramidase-like isoform X2, the lipid transport protein apolipophorin-III-like, the allergen icarapin, and UP LOC551098.

### Quantitative analysis

**Fig 2:**
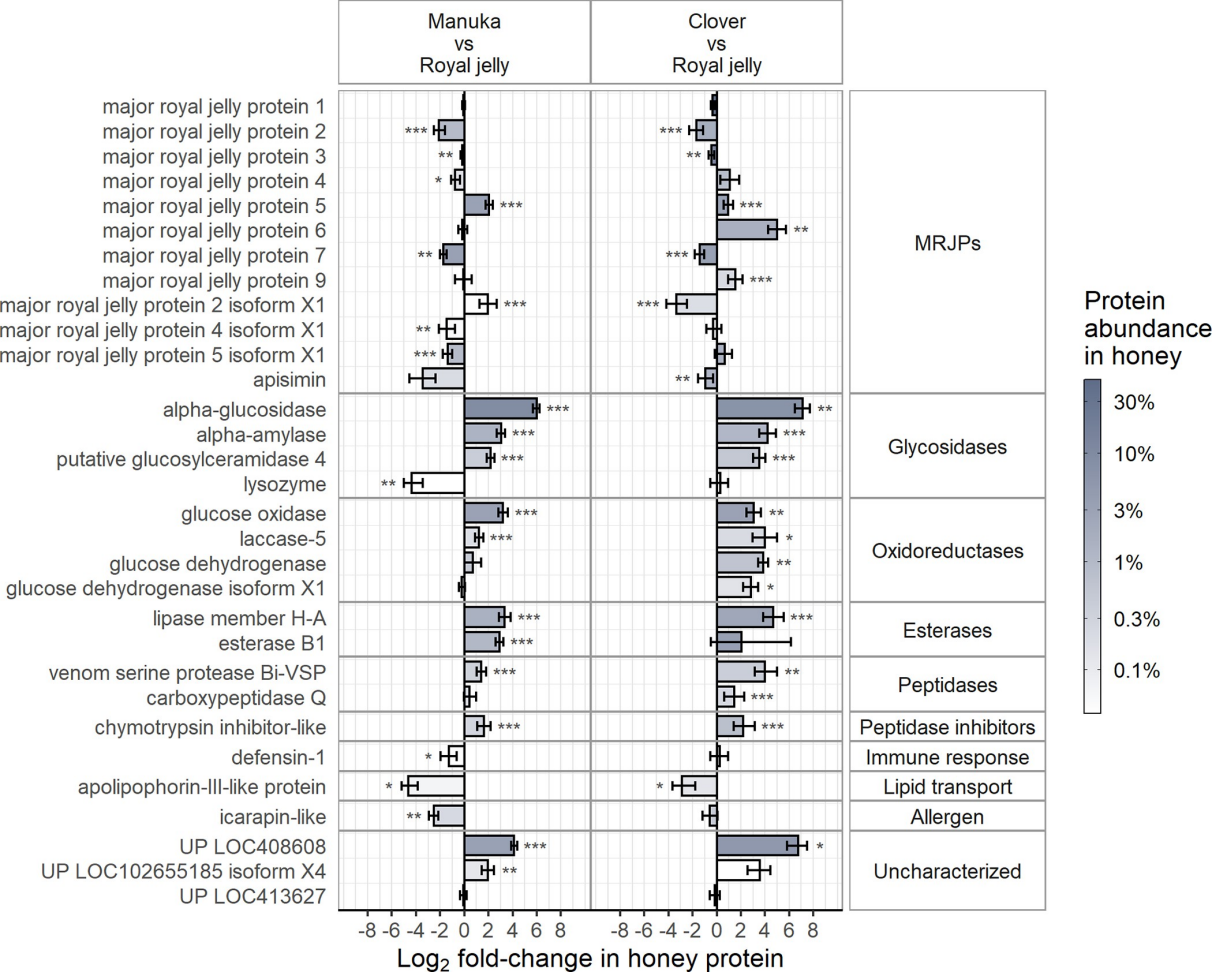
Comparative analyses of mānuka and clover honey to royal jelly by SWATH-MS. Quantitative comparisons of proteins identified by both analysis methods, expressed as the log_2_-fold change in sum of peak intensities for each honey protein compared to the corresponding protein found in royal jelly. Apisimin was added from the SWATH-MS analysis, giving 32 proteins in total. The shading of the bars represents the mean peptide intensity of protein in the honey samples (mānuka, n = 12; clover, n = 6, royal jelly n = 8). Significant differences are indicated by * *P* < 0.05, ** *P* < 0.01, *** *P* < 0.001.

Proteins identified in both analysis workflows (union of Fig 1B) were the focus of comparative quantitative analyses. There were 30 proteins identified by DIA-Umpire workflow and in both SWATH-MS comparisons. All 30 proteins were previously identified in the literature. Apisimin was also quantified by including a SWATH-MS window between m/z 315–321.4 to monitor the doubly charged peptide precursor TSISVK at m/z 317.69. The SWATH-MS quantitative data were analysed pairwise, comparing royal jelly to both mānuka and clover honey separately (Fig 2). Only peptides unique to a protein were included in these analyses to ensure that comparisons between groups were of peptide intensities from the same protein. In doing so, the sum of peak intensities became a less reliable measure of protein abundance. However, in contrast to the DIA-Umpire workflow, the same peptide coverage per protein was considered in each group for the ProteinPilot analysis. Regardless of accuracy, the inclusion of honey protein abundance data as shaded bars in Fig 2 helps to highlight the importance of some fold-change observations.

To look for patterns in relative MRJP abundance, all MRJP proteins were grouped and ordered. The remaining proteins were grouped by enzyme classification or biological role and ordered similarly. MRJP1 and 3 were most abundant in all groups and, therefore, little variation was observed at the Log_2_ scale. As MRJP7 was relatively high in royal jelly, there was 3.39-fold and 2.61-fold less MRJP7 in mānuka and clover, respectively. There was significantly more MRJP5 in the honey samples than in royal jelly. MRJP6 was 32.16-fold more abundant in clover than in royal jelly. In contrast, the difference was not significant between mānuka and royal jelly. Significantly less MRJP2 was found in both honeys compared to royal jelly. Generally, there were less of the remaining MRJPs in mānuka, and more in clover, than in royal jelly.

The non-MRJPs mostly comprised metabolising enzymes. Alpha-glucosidase was most abundant in this group with 63.2-fold and 126-fold more protein in mānuka and clover than in royal jelly, respectively. The UP LOC408608 was the next greatest in fold change followed variably by glucose oxidase, alpha-amylase, lipase member H-A, esterase B1, putative glucosylceramidase 4, and glucose dehydrogenase. These enzymes were also consistently found in previous studies in both honey and royal jelly. A consistent pattern was observed in both mānuka and clover honey, wherein the non-MRJPs were present in higher amounts than in royal jelly, except for glucosylceramidase-like isoforms, apolipophorin-III-like protein, and icarapin-like protein. Additionally, lysozyme and defensin-1 were 20-fold and 2.46-fold lower in mānuka honey than in royal jelly. Venom serine protease Bi-VSP and a chymotrypsin inhibitor-like protein were significantly more abundant in both honeys than in royal jelly, while carboxypeptidase Q was more abundant in clover only.

## Discussion

Shotgun proteomics and relative quantification by label-free SWATH-MS were used on numerous independent samples of mānuka honey, clover honey and royal jelly, to provide a comprehensive quantitative analysis and comparison of these honey varieties with royal jelly. The comparisons were made relative to the extracted protein component, while keeping in mind the large difference in absolute protein content between royal jelly and honey on a mass for mass basis.

Overall, the present study confirmed 52 proteins found previously while 24 were unique identifications. Several previous studies used equivalent or more sensitive instruments than was used here [16, 18, 32–34]. However, the methods, sample types and analyses varied to differing degrees. Most of the unique proteins in the SWATH-MS workflow were of relatively low abundance. Even so, some of these were actually more abundant in royal jelly than honey and of 10 unique proteins attributable to royal jelly in this way, only half had predicted signal peptides using SignalP 6.0 [50]. Similarly, two of 9 unique low abundance clover proteins had predicted signal peptides. While the absence of a signal sequence is not a guaranteed predictor of non-excretion, as other researchers have noted [33], substances produced in the hive are not free from contamination by other non-excreted bee proteins, and future proteomics studies will likely identify other unique, but extraneous, proteins. A recent study by Bong et al. [32] analysed mānuka honey using a similar instrument and software workflow to this study. There were 31 proteins common to both studies. However, there were 30 and 29 unique proteins respectively, in this study and the former. This comparison illustrates how differences can quickly arise with sample preparation methods (dialysis vs precipitation), LC run times (60 minutes vs 90 minutes) and particularly choice of database (RefSeq vs UniProtKB/TrEMBL). Nevertheless, from the perspective of this study the differences were trivial as many of the unique proteins were low in abundance, while those present in greater quantities were different isoforms of the major proteins.

We selected a group of robustly identified bee proteins also found previously in the literature for our quantitative analysis and comparisons. The difference in relative protein abundance of MRJPs was generally consistent between the honey types, with some exceptions. MRJP1 was the most abundant protein and was unchanged in both honeys compared to royal jelly. Likewise, MRJP2, 3, 5 and 7 levels changed to an equivalent degree in both honeys. MRJP transcript abundances are known to decrease from nurse bees to forager bees, but does not appear to cease entirely [51]. In royal jelly, MRJP1 is involved in larval development [52], likely in concert with the other MRJPs [53]. Control of development is obviously not required for adult bees in the hive, leading to a question of the purpose of MRJPs in honey. Forager bees are known to revert back to nurse bees if there are not enough nurse bees to service the needs of the hive [54]. The decrease in expression and low levels found in honey could be a result of ‘idle’ expression, allowing a quick reversion to the nurse bee phenotype when required. Alternatively, another role for MRJP1 involves binding 24-methylenecholesterol in an oligomeric MRJP1-apsimin structure [55]. This is an intriguing observation in light of work by Svoboda et al. [56] who describe the selective transfer of essential plant sterols to bee larvae in constant proportions via the brood food. The proportions of sterols measured in royal jelly by Svoboda [57] correlate with the proportions of abundant MRJPs in royal jelly determined from the ProteinPilot workflow when just the MRJPs are considered: MRJP1 54–60%, 24-methylenecholesterol 49–58%; MRJP3 20–23%, sitosterol 19–24%; MRJP7 9–12%, isofucosterol 10–16%; MRJP2 5–6%, campesterol 6–7%; MRJP5 1–2%, desmosterol 1–4%. This could be coincidence; however, plant pollen is highly variable in sterol composition and content, with 24-methylenecholesterol on average constituting 23% of total sterols, isofucosterol 21.5%, and sitosterol 20.7% [58]. Foraging different pollen sources may make up any deficit. However, Svoboda et al. [57] also measured the sterol content of whole nurse bees, which consisted of 27.3% 24-methylenecholesterol, 28.3% isofucosterol, and 25.7% sitosterol. This means that the hypopharyngeal or mandibular glands must allow entry into, and regulate exit from, secretory cells into a mostly aqueous substance. Protein sterol carriers are ubiquitous in nature, providing a means for transporting highly hydrophobic substances in polar mediums. A sterol carrier protein mechanism provides a simple explanation for sterol selectivity, the existence and consistent abundance of related MRJPs, and an efficient means for delivering constant proportions of sterols in the royal jelly diet of larvae.

One exception was MRJP6, which was significantly more abundant in clover honey than royal jelly. This observation supports the increased expression and translation of MRJP6 in the hypopharyngeal glands of forager bees [6, 51, 59]. However, the abundance in mānuka honey was equivalent to that in royal jelly, suggesting expression or translation of MRJP6 is not increased in bees that forage mānuka. Furthermore, the magnitude of the difference of MRJP6 between clover and royal jelly is equivalent to that found for other nectar metabolising proteins. Dobritzsch et al [51] suggest different transcription factors may control MRJP6 expression compared to the other MRJPs, and our data suggest this expression could be driven by a component within the type of nectar foraged that MRJP6 potentially metabolises, which is absent from mānuka honey. A measure of MRJP6 mRNA and protein abundance in the hypopharyngeal glands of mānuka foraging bees would test this hypothesis.

Oligomeric MRJP1 consists of a dimerised structure of two MRJP1 molecules bound to two 5 kDa apisimin molecules [55], which are known to polymerise at low pH [7, 60]. Previous proteomics studies of royal jelly and honey have identified apisimin using gel electrophoresis for separation [13, 14, 31]. Despite using 10 kDa dialysis membrane and relatively high pH buffer in this study, the MRJP1-apisimin dimer was still expected to be present in the prepared samples. Yet apisimin was not identified in the initial shotgun acquisition, and closer inspection of simulated tryptic digest revealed that a single peptide (TSISVK) would be detected with a doubly charged m/z ratio of 317.69. More recent HRMS studies of honeys [32–34] seemed unable to identify this peptide because survey scans were set from 350 m/z upwards. Difficulties with specifically identifying apisimin by MS have also been encountered previously [7, 61], even with chymotrypsin digestion [7]. We suggest using Glu-C as an alternative digest enzyme for studies focused on apisimin. For the present study, an additional window was included in the SWATH-MS protocol and the target apisimin peptide was detected with appreciable intensity in royal jelly and in both honey varieties. Our use of a narrow search database and inclusion of a short single peptide for identification and quantification of apisimin should be interpreted with caution, however.

Due to the quaternary structure, the molar ratio of MRJP1 to apisimin was expected to be 1:1. To estimate the ratio in each sample group, the proportional mean intensity was divided by peptide coverage. As a result, there was approximately 26-fold less apisimin than MRJP1 in mānuka, 4.9-fold less in clover, and 2.6-fold less in royal jelly. As the amount of MRJP1 was similar for each sample group in the SWATH-MS dataset, the discrepancy in ratios suggests it was not detected in mānuka, rather than being absent from the sample. It is likely that apisimin is glycated in mānuka honey, and peptide modifications must be known and specified to be detected. While glycations by MGO were included in the ProteinPilot search, the discrepancy suggests more complex glycations, or crosslinks to other peptides might occur, which could also affect trypsin cleavage of the single peptide, and, therefore, hamper detection in MS experiments.

Previous gene expression data has demonstrated upregulation of alpha-glucosidase in the heads of forager bees [62]. Here, glycosidases, oxidoreductases and esterases were far more abundant in honey compared to royal jelly. Nectar metabolism is required for production of honey. Therefore, some upregulation of enzymes was expected. However, glucosylceramidase-like isoform X2 was more abundant in royal jelly than in both honeys. When compared to the putative glucosylceramidase 4 upregulated in both honeys, similar functional domains are present, with BLAST alignment of 51% identity over 98% of the sequence. It is possible that in royal jelly there is a specific role for this glycosidase, having similar catalytic function but distinct substrate specificity. Several additional enzymes were also quantified in the SWATH-MS analysis (S1 Table). However, those represented in Fig 2 constitute the most important.

Two peptidases and a single chymotrypsin-like inhibitor were present in both honeys in higher amounts than in royal jelly. Roles in immune defense are likely as the venom serine protease contains a clip domain involved in regulating or localising the immune response [63]. The presence of the chymotrypsin-like inhibitor may help to prevent degradation of bee proteases and other proteins by exogenous proteases. The amount of defensin-1 was similar in clover honey and royal jelly, in agreement with previous studies showing similar expression of defensin-1 in the heads of nurse and forager bees [62]. It was also demonstrated via immunoblotting that different amounts of mature defensin-1 were present in different varieties of honey, yet it was unclear whether the difference was made up in each honey by unprocessed pro-form defensin-1. The processed state of defensin-1 in mānuka honey should not affect the measurements in our experiments due to all quantified peptides belonging to the mature peptide of defensin-1. Therefore, an equivalent amount of defensin-1 should have been observed in mānuka and royal jelly. This discrepancy may indicate that actual differences in expression of defensin-1 exist, depending on honey type, as noted for other proteins. As regulation of defensin-1 occurs via the Toll receptor pathway [64], expression should be linked with bacterial burden. In mānuka honey, bacterial burden is generally lower due to the presence of MGO, which may indirectly act to reduce Toll receptor signaling and hence expression of defensin-1. Other bactericidal substances in mānuka honey [65] would also contribute to this effect. These data support down-regulation of defensin-1 in mānuka honey. However, as with apisimin, undetectable glycation PTMs cannot yet be ruled out for the lack of bactericidal activity [62], or peptide abundance in these MS analyses. Additionally, hymenoptaecin was detected in mānuka by the SWATH analysis although the sum intensity was nearly 10-fold lower than defensin-1. Immune-stimulating functions have also been described for insect apolipoproteins [66, 67]. Apolipophorin-III-like protein was more abundant in royal jelly than in both honeys, reflecting the important role played in immune responses and lipid transport for developing larvae [68].

The Allergen Nomenclature database [69] lists 14 official bee allergens with GenBank [70] and UniProt identifiers. All have identical entries in RefSeq but only two of the three listed MRJP allergens were detected in this study, MRJP1 and MRJP9. Additionally, a commonly found ‘icarapin-like’ protein was identified which was assigned to accessions with similar sequences (NP_001012431.1 and XP_006563266.1) because the peptide coverage did not include sequence unique to either. The latter accession is equivalent to the Api m 10 sequence provided by the Allergen Nomenclature database, meaning this protein could alternatively be assigned as the icarapin variant 2 allergen. Additionally, two proteins were identified in the SWATH-MS workflow with very similar sequences to the acid phosphatase Api m 3, which have also been found in honey previously [33]. We did not find venom dipeptidyl peptidase 4 precursor nor MRJP8 in royal jelly as previously described [13, 16], nor did we find additional allergens that were previously found in honey [33, 34, 50], some of which appear to be isolated to single honey samples.

Three UPs were included in the quantitative comparison, while a further eight were identified in the SWATH-MS workflow (S1 Table). UP LOC102655185 is a serine-rich protein with extracellular signal sequence. This was far less abundant in all groups than the other UPs, yet there was at least 4-fold more in honey than in royal jelly. BLAST alignment with non-redundant sequences matched closely to several mucins, which may indicate a digestive tract origin such as the crop. UP LOC413627 was similarly abundant in all three groups, but 6–7.6-fold less abundant than UP LOC408608. BLAST search results indicate UP LOC413627 has a regucalcin-like calcium binding domain, and no signal peptide, indicating an intracellular origin.

UP LOC408608 has been identified several times previously in both honey and royal jelly [9, 14, 16, 18, 33, 71, 72], yet has no clear function assigned. To our knowledge, this is the first time it has been quantitatively compared in the two bee products. Proteomic profiles of several bee glands found that this protein is likely secreted from the postcerebral and thoracic glands, rather than the hypopharyngeal gland [71]. Our data support those observations by the relative absence of UP LOC408608 from royal jelly and relatively high amount found in both mānuka and clover honey. BLAST alignment provided six hits–UPs from five different *Apis* species and one UP from the plant pathogen *Xanthomonas hortorum*–providing no indications of the function. Therefore, the lack of similarity to known sequences from any other genus (except species *X*. *hortorum* alone), abundance equivalent to nectar metabolising proteins, and the relative absence from royal jelly together indicate that this protein has a unique nectar-specific or honey-specific role.

## Conclusion

Quantitative data in the present study further confirms that expression at the protein level changes as bees develop through the worker caste, with subtle changes in MRJP expression, and extensive changes in proteins with enzymatic function. Differences in the abundance of MRJP6 between mānuka and clover honey suggest a potential effect of foraged nectar type on expression. The abundance of MRJP6 in clover honey and uncharacterised protein LOC408608 in both honeys was similar to that of other nectar metabolising enzymes suggesting that these proteins potentially have a catalytic role in nectar processing.

## Supporting information

S1 Table Venn diagram data. Data used to compile Fig 1 Venn diagrams.
(XLSX)

S1 Data Fig 2 data. (XLSX)

S2 Data Protein Prophet data. (XLSX)

S3 Data DIA-Umpire data. (XLSX)

S4 Data ProteinPilot data. (XLSX)
